# Acute respiratory infection and associated factors among young children presenting to hospital in Sierra Leone

**DOI:** 10.1093/inthealth/ihag057

**Published:** 2026-06-13

**Authors:** Morie Vandi, Troy D. Moon, Gustavo Amorim, John S. Schieffelin, Robert J. Samuels

**Affiliations:** 1 Ministry of Health and Sanitation, Freetown, Sierra Leone; 2 London School of Hygiene and Tropical Medicine, London, United Kingdom; 3 Department of Pediatrics, Division of Pediatric Infectious Diseases, Tulane University School of Medicine, New Orleans, LA, United States; 4 Department of Tropical Medicine and Infectious Diseases, Tulane University Celia Scott Weatherhead School of Public Health and Tropical Medicine, New Orleans, LA, United States; 5 Department of Biostatistics, Vanderbilt University Medical Center, Nashville, TN, United States; 6 Kenema Government Hospital, Ministry of Health and Sanitation, Kenema, Sierra Leone

**Keywords:** acute respiratory infection, pediatric, respiratory virus, risk factors, Sierra Leone

## Abstract

**Objectives:**

Acute respiratory infections (ARIs) are a leading cause of infant morbidity and mortality worldwide, with a substantial burden in Sierra Leone. Although maternal, infant, and environmental factors influence ARI susceptibility, their role in disease severity remains unclear.

**Methods:**

We conducted prospective surveillance of children aged <24 months admitted to Kenema Government Hospital from October 2020 to October 2021. Eligible children were enrolled within 48 hours of admission and had at least one acute respiratory symptom. Nose and throat swabs were collected and tested using multiplex reverse-transcription PCR. Statistical analyses were performed in R.

**Results:**

Among 502 enrolled children, 376 (74.9%) had ≥1 viral pathogen detected by PCR. Younger age was associated with viral detection (*P* = .009). Global chronic malnutrition was less common among virus-positive than virus-negative children (36.5% vs 48.0%, *P* = .030). Infants aged 0–6 months and those breastfed for shorter durations experienced more severe disease. Each additional month of breastfeeding reduced the odds of a higher ARI severity score by 6% (OR 0.94, *P* = .004).

**Conclusion:**

Viral ARIs are a major health challenge among young children in Sierra Leone. Breastfeeding duration and nutritional status were important modifiable risk factors associated with disease severity, highlighting opportunities for interventions.

## Introduction

Acute respiratory infections (ARIs) are a significant public health concern globally, particularly among infants and young children. Respiratory infections contribute to 6% of the total global disease burden, with 6.6 million children aged <5 years dying each year, mostly in low-income countries with limited healthcare resources.^1,2^ Sierra Leone, a country in West Africa, bears a substantial burden of ARIs, with high rates of under-five mortality attributed to respiratory infections.^3^ The Kenema Government Hospital (KGH) in Eastern Province, Sierra Leone, is a critical regional referral hospital that experiences a high prevalence of pediatric ARIs.^4^ Understanding the factors influencing the severity of ARIs in this population is crucial for implementing effective preventive measures and improving healthcare outcomes.

This study was conducted from October 2020 to October 2021, during the COVID-19 pandemic. In Sierra Leone, reported COVID-19 incidence was lower than in many settings.^5^ Public health measures implemented during this period, including mask-wearing, restrictions on gatherings and travel, and periodic school disruptions, may have influenced the circulation and seasonality of common respiratory viruses and affected healthcare-seeking behavior.

Several preventable sociocultural, demographic, and environmental risk factors predispose children to severe ARIs.^6^ However, these factors have not been well investigated in children living in Sierra Leone. Understanding the impacts of these risk factors is essential for developing effective strategies to reduce the severity of ARIs in this vulnerable population.

Potential determinants for the risk of ARIs in infants include maternal risk factors involving various aspects of maternal health, behavior, and socioeconomic status. For example, insufficient prenatal care, including limited access to antenatal visits, inadequate maternal vaccination, and inadequate health education for mothers, have been shown to increase the risk of ARIs in infants.^7-9^ Other maternal factors such as age, smoking, socioeconomic status, education level, breastfeeding practices, and occupation have been attributed to the susceptibility to and severity of ARIs in infants.^10-12^ In addition to maternal factors, other risk factors such as infant age, gender, nutritional status, whether the ARI is bacterial or viral, and co-infections should be considered as potential drivers of ARI outcomes.^1,8,9^ Finally, environmental factors such as exposure to indoor air pollution, such as second-hand cigarette smoke and the use of indoor burning stoves for cooking, are also associated with a higher likelihood of respiratory infections in children.^13,14^ It is important to distinguish factors associated with the presentation of ARIs from those driving disease severity among hospitalized children, a distinction for which data in Sierra Leone remain limited. Although viral pathogens are frequently detected among hospitalized children with ARIs in low- and middle-income countries (LMICs), viral presence alone does not explain the variability in clinical severity. Clarifying host and contextual factors associated with (i) viral detection, and (ii) disease severity are therefore complementary goals critical for informing targeted prevention and clinical management.

Accordingly, the current study’s objectives were (i) to estimate the proportion of children aged < 24 months hospitalized with acute respiratory symptoms at KGH who had a respiratory virus detected by PCR at enrollment; (ii) to identify maternal, infant, and contextual factors associated with viral detection by PCR; and (iii) to identify factors associated with increased ARI severity among hospitalized children. This study builds on the viral surveillance platform described in.^15^

## Materials and methods

### Study design and population

We conducted a prospective surveillance study of children aged <24 months admitted to KGH from October 2020 to October 2021. Our methods are published elsewhere.^15^ Eligible participants had to have been admitted within 48 hours of study enrollment and exhibited at least one acute respiratory symptom, defined as a cough lasting <14 days; breathing difficulty characterized by chest wall retractions and age-specific tachypnea (> 50 breaths/min for infants aged 0–12 months and >40 breaths/min for those aged 13–23 months); and/or nasal flaring. The participants were enrolled only once.

### Study setting

The study took place at KGH, situated in Kenema District of the Eastern Province in Sierra Leone. This regional hospital has a catchment area of ~670 000 residents, with a capacity of 350 beds.^16^ The area is endemic to various tropical diseases, such as Lassa fever, malaria, yellow fever, TB, and intestinal parasites.^17^ The pediatric ward at KGH provides free healthcare to children aged <5 years, admitting ~400 children monthly. Guidelines for pediatric patient management and triage at the point of hospital admission adhere to the Emergency Triage Assessment and Treatment plus (ETAT+) protocol for patient care.^15^

### Recruitment

Upon admission, children presenting with acute respiratory symptoms were first evaluated by the ward’s emergency care nurses. If they met the inclusion criteria, designated study staff approached the parents or legal guardians for enrollment, with a daily maximum enrollment of three new patients per day. Enrollment followed a convenience sampling approach. On days when more than three eligible children were admitted, participants were enrolled on a first-identified, first-consented basis during working hours between 6 a.m. and 6 p.m. from Monday to Friday.

### Data collection and severity score

The study nurses collected data using a paper-based questionnaire. These data were later transferred to a secure, tablet-based online Research Electronic Data Capture (REDCap) database. Parents or legal guardians answered a set of questions using a standardized case report form covering seven main domains: introduction and screening; demographics; social history; current illness; diet/nutrition; physical examination; and laboratory results. At enrollment, within 48 hours of hospital admission, the severity of participants’ respiratory symptoms was assessed using a modified version of the bronchiolitis severity score developed by Tal and colleagues.^18,19^ Children were then categorized as mild (≤6), moderate,^7-9^ or severe^10, 11^ based on the severity of their symptoms at the time of data entry. The components of the modified Tal severity score and the cut-off values used to classify mild, moderate, and severe disease are provided in Supplementary Table 1. Chart reviews were conducted during admission and again at discharge to document demographic details, medical and medication history, and the clinical course during hospitalization. To maintain data accuracy, data quality control was performed by reviewing all completed paper-based study instruments and verifying the precision of the information entered into the electronic database. The severity score reflected clinical status at the time of initial study assessment and was applied uniformly to all enrolled children meeting ARI criteria; we acknowledge that symptom evolution and treatment initiation prior to assessment may have influenced severity classification.

### Laboratory procedures

Nose and throat swabs were collected from participants at enrollment and promptly placed in viral transport medium. To preserve anonymity, each sample was labeled with the participant’s study identification number. These samples were then stored at −80°C at KGH until they were shipped on dry ice (in November 2021) to Vanderbilt University Medical Center in Nashville, Tennessee, USA, for testing using total nucleic acid (TNA) extraction, followed by reverse-transcription PCR. TNA was purified from aliquoted specimens using the QIAamp 96 Virus QIAcube HT Kit and QIAcube HT automated extraction system (QIAGEN, Germantown, MD, USA) and maintained at −80°C until analysis. TNA extracts were tested for the following pathogen targets using the NxTAG Respiratory Pathogen Panel + SARS-CoV-2 (Luminex, Austin, TX, USA): adenovirus (AdV); human endemic coronaviruses (hCoV) 229E, HKU1, NL63, and OC43; SARS-CoV-2; human bocavirus (hBoV); human metapneumovirus (hMPV); influenza A and subtypes 2009 H1N1, H1, and H3; influenza B and influenza C; parainfluenza (PIV) 1–4; respiratory syncytial virus (RSV) A and B; human rhinovirus/enterovirus (HRV/EV); *Chlamydophila pneumoniae*; and *Mycoplasma pneumoniae*.^15^

### Data analysis

Data tabulation and analysis was conducted using Microsoft Excel 365 version 2307 and R version 4.3.1. Continuous data were summarized in terms of medians and IQR; categorical variables were summarized by counts and proportions. In line with the STrengthening the Reporting of OBservational studies in Epidemiology (STROBE) recommendations, outcomes and exposures were defined *a priori*. The primary outcomes were (i) viral detection by PCR (binary outcome), and (ii) ARI severity score at enrollment (ordinal outcome). Key exposures included child age, nutritional status (global acute and chronic malnutrition), breastfeeding duration, maternal age and education, household smoking exposure, and birthweight. Global acute malnutrition (GAM) was defined as a weight-for-height z-score of < −2 and/or bilateral pitting oedema, while global chronic malnutrition (GCM) was defined as a height-for-age z-score of < −2, according to WHO Child Growth Standards.^20^ For data analysis, specific statistical methods were applied and tailored to different types of variables and study goals. The non-parametric Wilcox–Mann–Whitney test was used for comparing continuous variables, and chi-square tests for categorical variables. To explore factors associated with an increased likelihood of viral infection, a multivariable logistic regression analysis was performed. An ordinal regression model was used to assess factors linked to severity scores. All multivariable regression models had variables selected *a priori*, and adjusted for sex, birthweight, GAM, GCM, breastfeeding duration, whether the patient was sick in the last 2 weeks, whether a household member smokes, mother’s education, and mother’s age. *A priori* variables were selected based on published literature from LMIC settings, biological plausibility, and data availability. Models were fully specified rather than selected using stepwise procedures to reduce data-driven overfitting.

Because breastfeeding duration and age were highly correlated (Pearson correlation = 0.92), we decided not to include the two variables simultaneously in the regression models to avoid issues with multicollinearity. To address this collinearity, multivariable analyses assessing disease severity were restricted to children aged 6–24 months; age was therefore not included as a covariate in the ordinal regression model. Instead, for the multivariable analysis, we focused only on patients aged 6–24 months. We acknowledge that restricting analyses to children aged 6–24 months does not fully resolve collinearity between age and breastfeeding duration and excludes infants aged <6 months, who represent a particularly vulnerable group for severe ARIs. Viral detection was treated as a binary exposure variable (virus detected vs not detected) rather than stratified by pathogen. This analytic approach was chosen *a priori* to align with the primary study objective of identifying host-level maternal and infant risk factors associated with ARI presence and disease severity, rather than pathogen-specific epidemiology. Detailed pathogen distribution, co-infection patterns, seasonality, and SARS-CoV-2–specific findings from this study population have been reported previously.^15^

### Sample size

No formal sample size estimation was performed, as this analysis used all available participants from an ongoing surveillance platform.

## Results

### Characteristics of the study population

**Figure 1:**
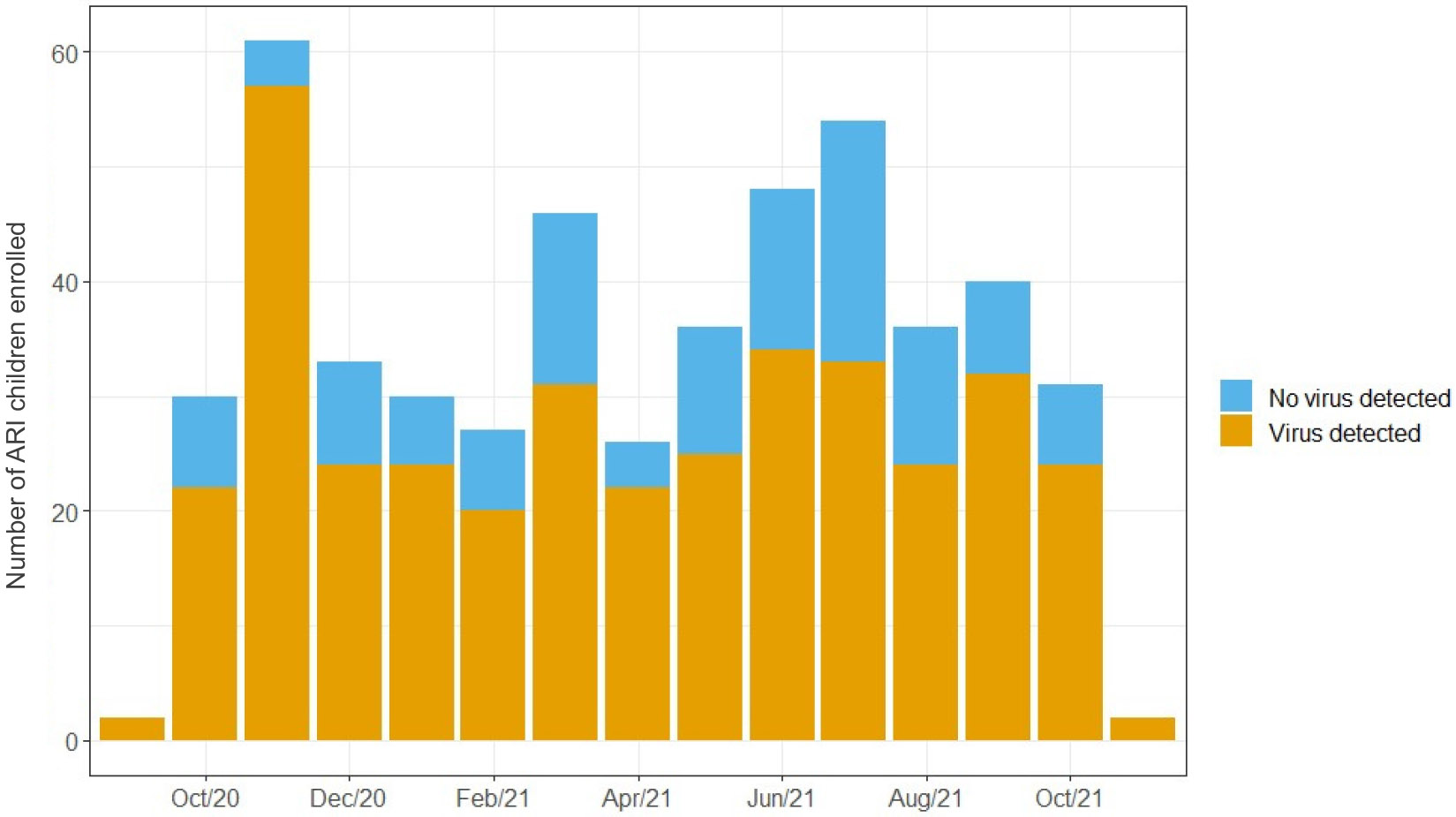
Prevalence of ARI during the study period.

From October 2020 to October 2021, a total of 502 children aged <24 months were admitted to KGH with respiratory symptoms (Table 1). Of these, 68% (*n* = 341) were aged 0–12 months and 52.4% were males. The median birthweight was 3.0 (IQR 2.80–3.40) kg. More than 35% of all children were considered to have either GAM or GCM. Maternal education was generally low, with ~47% of all mothers having achieved only some primary education or less. A history of breastfeeding among our study population was nearly universal (97.4%), with a median length of breastfeeding for 8 (5.0–13.0) months. The number of children with a virus detected at enrollment is displayed in Figure 1.

### Characteristics of virus-positive participants

**Figure 2:**
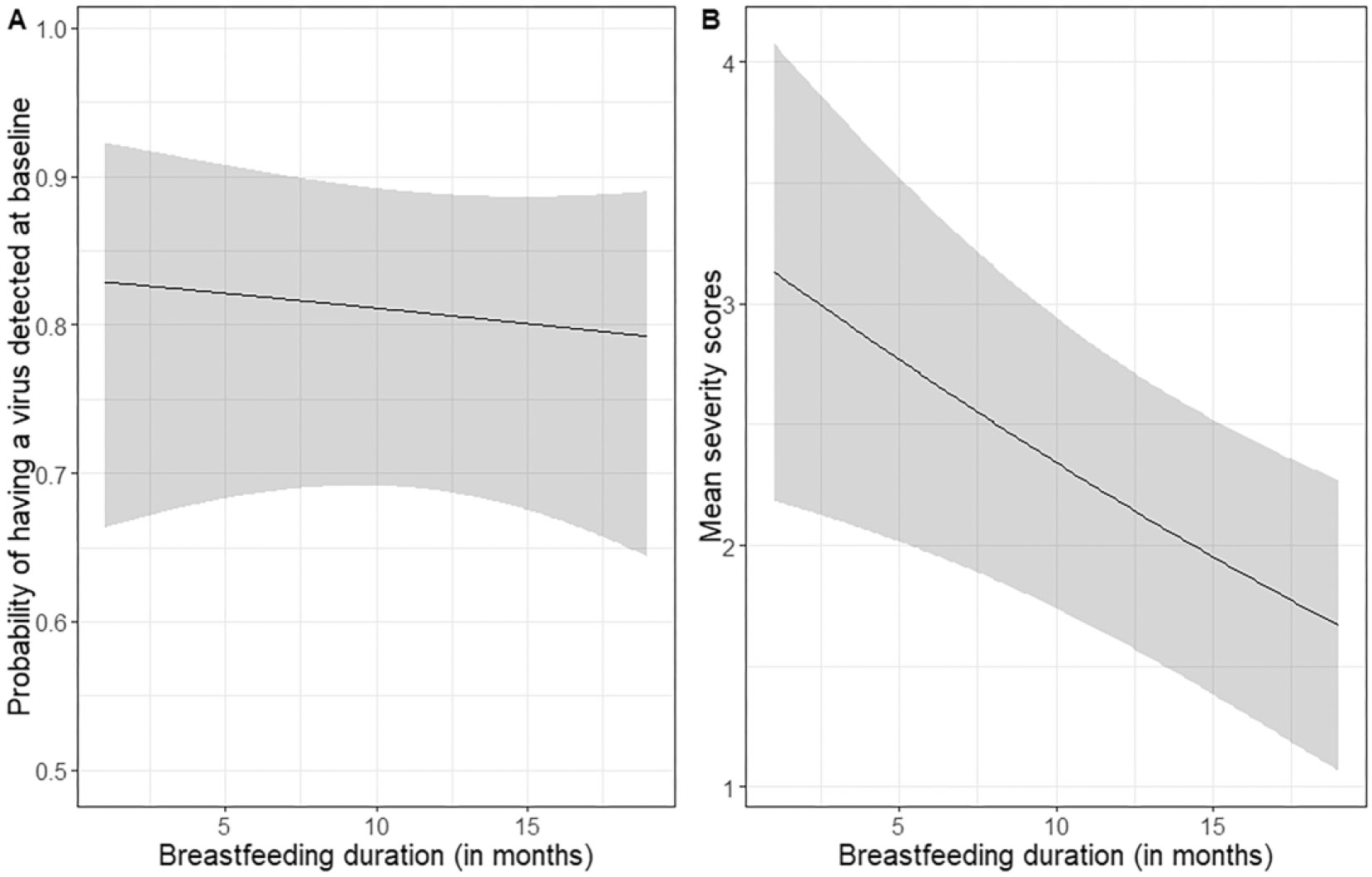
Association of breastfeeding duration (in months) with the outcomes of interest, among children 6 to 24 months old. Panel A) shows the probability of having virus detected at baseline in terms of breastfeeding duration and Panel B) shows the impact of breastfeeding duration on the mean severity score. All other variables from the multivariable regression models were set to their reference level (categorical variables) or to their median values (continuous variables).

A total of 376 children (74.9%), admitted with symptoms of ARIs, were found to have at least one viral pathogen detected on nose or throat swab PCR (Table 1). Virus-positive participants were younger than virus-negative participants at the time of enrollment: a median age of 8.1 (4.5–13.7) months compared with 10.5 (6.2–15.6) months in the virus-negative group (*P* = .009). Although SARS-CoV-2 was included in the testing panel, it accounted for a very small proportion of viral detections, consistent with low pediatric COVID-19 hospitalization rates in Sierra Leone during the study period.^5^ The proportion of participants with GCM was lower in virus-positive compared with virus-negative participants (36.5% vs 48.0%, *P* = .030). Children admitted to hospital with an ARI for whom a virus was detected at enrollment generally had a shorter history of breastfeeding duration: 7.0 (4.0–12.0) vs 9.5 (5.0–14.0) months (*P* = .025). Because breastfeeding duration was highly correlated with the child’s age (Pearson correlation = 0.92), we ran a stratified analysis focusing on patients aged 6–24 months, that is, among those who had the opportunity to complete the recommended 6 months of exclusive breastfeeding. Among this subgroup (*n* = 341), children diagnosed with moderate/severe GCM had lower odds of viral detection (OR 0.55, 95% CI: 0.33–0.92, *P* = .023). Breastfeeding duration was associated with viral detection in unadjusted analyses; however, this association was no longer statistically significant after age-restricted multivariable analyses (Table 2, Figure 2).

### Severity of ARIs in infants

Within 48 hours of hospital admission, children’s respiratory symptoms were scored from 0 to 11 then classified into mild, moderate, and severe categories (Table 3). When analyzing the entire sample, we noticed that younger children were more likely to have severe symptoms: the median ages for mild, moderate, and severe disease severity, respectively, were 10.2 (5.9–15.6), 6.4 (3.7–11.1), and 5.2 (2.1–8.8) months (*P* < .001). Moderate and severe respiratory symptoms were seen in a higher proportion among children aged 0–6 months compared with older children (*P* < .001). Furthermore, children who had had a shorter duration of breastfeeding were more likely to have more severe respiratory symptoms (*P* < .001).

Among breastfed children, only 5% (25 out of 489) exhibited severe ARI symptoms, in contrast to 15% in the non-breastfed group (2 out of 13). Although the proportion of children with a virus detected at the time of enrollment varied across severity groups, the presence of a virus had no statistical impact on disease severity classification upon hospital admission at the 5% level (*P* = .093).

Given the strong collinearity between child age and breastfeeding duration (Pearson correlation coefficient = 0.92), both variables were not included simultaneously in multivariable models. Although non-breastfed children had a numerically higher proportion of severe ARIs, this difference did not reach statistical significance. A multivariable ordinal regression, which treats severity score as a continuous ordinal variable, was performed to identify factors associated with increased disease severity (Table 4). To address this, severity analyses were restricted to children aged 6–24 months (*n* = 341), ensuring that all included participants had the opportunity to complete the WHO-recommended 6 months of exclusive breastfeeding. Longer duration of breastfeeding was protective against increased disease severity; each additional month of breastfeeding duration reduced the odds of having an increased severity score by 6% (OR 0.94, 95% CI: 0.89–0.98, *P* = .004).

## Discussion

To the best of our knowledge, this study provides the first hospital-based assessment of viral ARI severity among children aged <24 months in Sierra Leone and is the first conducted during the COVID-19 pandemic, offering rare context-specific evidence from a low-resource setting. Using hospital-based surveillance at KGH from October 2020 to October 2021, we examined (i) the proportion of hospitalized children with ARIs who had at least one respiratory virus detected by PCR; (ii) host-level factors associated with viral detection; and (iii) host-level determinants of disease severity assessed within 48 hours of admission across all enrolled children, regardless of viral detection status. Approximately three-quarters of children hospitalized with ARIs had at least one respiratory pathogen detected by multiplex PCR at enrollment, consistent with reports from LMICs, where viral pathogens account for 50%–80% of pediatric ARI hospitalizations, particularly in sub-Saharan Africa.^21-23^ Detailed pathogen-specific epidemiology, including distribution, co-infections, and seasonality, has been reported separately in a companion publication and was not re-analyzed here to avoid duplication.^15^ Importantly, these data provide context-specific evidence from Sierra Leone during the COVID-19 pandemic, a period when nonpharmaceutical interventions and altered healthcare-seeking behaviors may have influenced respiratory virus circulation and hospital admission patterns,^5^ further underscoring the substantial burden of viral ARIs in resource-limited settings. In this analysis, viral pathogens were aggregated rather than examined individually. While this approach aligns with the study’s focus on host-level determinants of disease severity, it may obscure pathogen-specific effects. It is likely that observed associations with severity are driven predominantly by viruses such as HRV/EV (28.2%) and RSV (19.5%), which were the most prevalent pathogens in this population (Supplementary Figure 1).^15^ Conversely, SARS-CoV-2 contributed minimally (3.9%) to overall detections, reflecting low pediatric COVID-19 hospitalization rates in Sierra Leone during the study period.

Because breastfeeding duration and child age were highly correlated, we employed an age-restricted analytic strategy to minimize confounding. When restricting the analysis to children aged 6–24 months, breastfeeding duration was no longer associated with viral detection in multivariable analyses, suggesting that this association in the full sample was largely driven by the confounding effect of age. Infants aged <12 months had higher odds of having a virus detected compared with older children, consistent with literature that highlights immature immune systems and high exposure to respiratory pathogens in early infancy as key risk factors.^24^ The attenuation of the breastfeeding association after age restriction suggests that the unadjusted association between breastfeeding duration and viral detection may partly reflect the confounding effect of age rather than a direct biological effect of breastfeeding on susceptibility to viral infection. Younger age and poor nutritional status were associated with greater ARI severity in univariable analyses, although the association with GCM did not persist after adjustment, despite its well-established role in impairing immune function.^25^ Similarly, younger age in our study was associated with higher disease severity scores, in line with previous findings that younger infants are more prone to severe ARIs due to immature immunity and smaller airway sizes.^26^ Gender was not associated with disease severity in our study, consistent with other studies that reported no significant differences in ARI severity between boys and girls.^27^

Of the maternal factors analyzed (age, education, and breastfeeding practices), breastfeeding duration had an association with disease severity. Each additional month of breastfeeding in children aged >6 months reduced the odds of severe ARI symptoms by 6% (OR 0.94, 95% CI: 0.89–0.98). These findings align with studies demonstrating that longer breastfeeding duration confers a dose-dependent protective effect against severe respiratory infections.^28^ Breastfeeding is well documented to enhance immune defenses by transferring maternal antibodies, reducing susceptibility to viral infection.^29^ These findings support the broader evidence base for the protective role of continued breastfeeding beyond 6 months of age and highlight the need to sustain breastfeeding support in this population. Although this study was restricted to children aged 6–24 months and did not analyze the impact of breastfeeding in the first 6 months of life, the findings are consistent with WHO recommendations for continued breastfeeding up to 2 years or beyond. Although maternal education was included in multivariable models, it did not retain statistical significance after adjustment, even although the available literature links limited maternal health literacy to delayed healthcare-seeking behaviors and suboptimal infant care practices.^30^ Socioeconomic status and smoking, although not analyzed in our study, are well-documented risk factors for severe ARIs in LMICs.^31^

Exposure to indoor cooking smoke and household tobacco smoke was not associated with ARI severity, although tobacco exposure was more frequent among severe cases and the study was underpowered to detect small effects. This contrasts with prior evidence linking indoor air pollution to poorer respiratory outcomes^32^ and suggests the need for more detailed assessment of ventilation and exposure intensity in this setting.

### Implications

These findings have important implications for clinical practice, public health, and research in low-resource settings. Clinically, improved risk stratification at enrollment may enhance triage and antibiotic stewardship, given the high burden of viral ARIs among hospitalized children. From a public health perspective, strengthening child nutrition programs and sustained support for continued breastfeeding beyond 6 months remain priorities. Although maternal education did not independently predict severity in adjusted analyses in this study, broader community-level investments in maternal health literacy remain important and merit evaluation in future longitudinal studies. Finally, future research should prioritize longitudinal, community-based studies to better disentangle the contributions of age, breastfeeding, nutrition, and specific pathogen types to ARI severity.

### Limitations

This study has several limitations. Enrollment was convenience-based and capped at three children per day, potentially introducing selection bias, and interviews conducted at hospital admission may have resulted in recall bias and inaccuracies in self-reported data. Severity scoring within 48 hours of admission may have been influenced by symptom evolution or early treatment and may not fully capture peak disease severity, as some children with ARIs may improve rapidly and be discharged within this period. Strong collinearity between age and breastfeeding duration required restricting severity analyses to children aged 6–24 months, excluding younger infants at the highest risk of severe ARIs and limiting generalizability to this group. The single-hospital study setting may not fully capture the diversity of maternal and infant risk factors across Sierra Leone, although KGH is broadly representative of similar public facilities; caution is therefore warranted when extrapolating findings to community-based or high-income settings. Residual confounding cannot be excluded. PCR-based viral detection does not establish causality and may reflect recent or concurrent infection, and the lack of comprehensive bacterial diagnostics and pathogen-specific severity analyses may have limited etiological interpretation.

## Conclusions

This study highlights the high prevalence of viral ARIs among children aged <24 months admitted to KGH with respiratory symptoms. In adjusted analyses, longer breastfeeding duration among children aged 6–24 months was associated with lower ARI severity scores, while associations with age and nutritional status were attenuated. These findings provide valuable insights for clinicians and public health policymakers, particularly in resource-limited settings such as Sierra Leone, where targeted interventions (maternal education, enhancing nutritional programs, and addressing environmental risk factors) could substantially reduce the burden of ARIs. Future research should focus on longitudinal studies to explore causal relationships and investigate the role of co-infections, environmental exposures, and access to care in shaping ARI outcomes.

## Supplementary Material

Supplementary Figure legend

Supplementary Table 1

Supplementary Figure 1

Supplementary ismaterial is available at International Health online.

## Figures and Tables

**Table 1:** Characteristics of children aged <24 months hospitalized at KGH with acute respiratory symptoms by respiratory virus detection.

Characteristic	Virus-negative (*n* = 126)	Virus-positive (*n* = 376)	Total (*n* = 502)	*P*-value
Child age (months), median [IQR]	10.5 [6.2; 15.6]	8.1 [4.5; 13.7]	8.6 [4.9; 14.2]	.009
Child age group (months)				.089
0–6	31 (24.6%)	137 (36.4%)	168 (33.5%)	
7–12	47 (37.3%)	126 (33.5%)	173 (34.5%)	
13–18	29 (23.0%)	73 (19.4%)	102 (20.3%)	
19–24	19 (15.1%)	40 (10.6%)	59 (11.7%)	
Child gender				.605
Male	63 (50.0%)	200 (53.2%)	263 (52.4%)	
Female	63 (50.0%)	176 (46.8%)	239 (47.6%)	
Birthweight (kg), median [IQR]	3.0 [2.7; 3.3]	3.0 [2.8; 3.4]	3.0 [2.8; 3.4]	.344
GAM^a^				.381
Moderate/severe	51 (41.1%)	135 (36.2%)	186 (37.4%)	
GCM^b^				.030
Moderate/severe	60 (48.0%)	137 (36.5%)	197 (39.4%)	
Mother’s age (years), median [IQR]	25.0 [21.0; 28.0]	24.0 [21.0;28.0]	24.5 [21.0; 28.0]	.655
Mother’s education				.338
No education	48 (38.1%)	118 (31.4%)	166 (33.1%)	
Primary education	17 (13.5%)	52 (13.8%)	69 (13.7%)	
Secondary education	53 (42.1%)	165 (43.9%)	218 (43.4%)	
Some college/university	8 (6.35%)	41 (10.9%)	49 (9.76%)	
BF^c^ duration (months), median [IQR]	9.5 [5.0; 14.0]	7.0 [4.0; 12.0]	8.0 [5.0; 13.0]	.025
History of BF				.533
Yes	124 (98.4%)	365 (97.1%)	489 (97.4%)	
No	2 (1.59%)	11 (2.93%)	13 (2.59%)	
Household tobacco smoke exposure				1.000
Yes	50 (39.7%)	149 (39.6%)	199 (39.6%)	
No	76 (60.3%)	227 (60.4%)	303 (60.4%)	
Household has indoor burning stove				1.000
Yes	8 (6.35%)	24 (6.4%)	32 (6.8%)	
No	118 (93.7%)	352 (93.6%)	470 (93.6%)	

a GAM = weight for height z-score < −2.

b GCM = height for age z-score < −2.

c BF = breastfeeding.

**Table 2:** Multivariable logistic regression to assess factors associated with being virus-positive at baseline, restricted to patients aged 6–24 months.

Predictor	OR (95% CI)	*P*-value
Sex: male	1.05 (0.64; 1.73)	.854
Birthweight	1.00 (0.61; 1.64)	.993
Moderate/severe GAM	0.75 (0.45; 1.24)	.258
Moderate/severe GCM	0.55 (0.33; 0.92)	.023
BF^a^ duration (per 1 month increase)	0.99 (0.93; 1.04)	.646
Sickness reported in the last 2 weeks: yes	0.78 (0.46; 1.33)	.366
Mother’s education: secondary or higher	1.22 (0.74; 2.01)	.436
Exposure to household tobacco smoke	1.13 (0.69; 1.88)	.626
Mother’s age	1.01 (0.96; 1.05)	.719

Sig: *P*-value at 5% level of significance.

a BF = breastfeeding.

**Table 3:** Summary description of severity of respiratory symptoms at admission.

Characteristic	Disease severity			
	Mild (*n* = 325)	Moderate (*n* = 150)	Severe (*n* = 27)	*P*-value
Child age (months), median [IQR]	10.2 [5.9; 15.6]	6.4 [3.7; 11.1]	5.2 [2.10; 8.84]	< .001
Child age group (months)				< .001
0–6	83 (25.5%)	70 (46.7%)	15 (55.6%)	
7–12	49 (15.1%)	9 (6.00%)	1 (3.70%)	
13–18	115 (35.4%)	50 (33.3%)	8 (29.6%)	
19–24	78 (24.0%)	21 (14.0%)	3 (11.1%)	
Child gender				.220
Male	175 (53.8%)	71 (47.3%)	17 (63.0%)	
Female	150 (46.2%)	79 (52.7%)	10 (37.0%)	
Virus: positive	235 (72.3%)	122 (81.3%)	19 (70.4%)	.093
Birthweight (kg), median [IQR]	3.0 [2.8; 3.4]	3.0 [2.8; 3.4]	3.0 [2.9; 3.4]	.964
GAM^a^				.382
Normal	207 (64.3%)	90 (60.8%)	14 (51.9%)	
Moderate/severe	115 (35.7%)	58 (39.2%)	13 (48.1%)	
GCM^b^				.015
Normal	186 (57.4%)	104 (69.8%)	13 (48.1%)	
Moderate/severe	138 (42.6%)	45 (30.2%)	14 (51.9%)	
Mother’s age (years), median [IQR]	24.0 [21.0; 28.0]	25.0 [21.0; 28.0]	27.0 [21.5; 29.0]	.495
Mother’s education				.070
Primary or less	153 (47.1%)	64 (42.7%)	18 (66.7%)	
Secondary and higher	172 (52.9%)	86 (57.3%)	9 (33.3%)	
BF^c^ duration (months), median [IQR]	9.0 [6.0; 14.0]	6.0 [3.0; 10.0]	5.0 [2.0; 9.0]	< .001
History of BF				.533
Yes	317 (97.5%)	147 (98.0%)	25 (92.6%)	
No	8 (2.46%)	3 (2.00%)	2 (7.41%)	
Household tobacco smoke exposure				.410
Yes	127 (39.1%)	58 (38.7%)	14 (51.9%)	
No	198 (60.9%)	92 (61.3%)	13 (48.1%)	
Household has indoor burning stove				1.000
Yes	21 (6.5%)	10 (6.7%)	1 (3.7%)	
No	304 (93.5%)	140 (93.3%)	26 (96.3%)	

a GAM = weight for height z-score < −2.

b GCM = height for age z-score < −2.

c BF = breastfeeding.

**Table 4:** Multivariable ordinal regression to assess the impact of each variable on disease severity, restricted to patients aged 6–24 months.

Predictor	OR (95% CI)	*P*-value
Sex: male	0.90 (0.60; 1.33)	.584
Birthweight	1.15 (0.77; 1.72)	.484
Moderate/severe GAM	1.25 (0.84; 1.87)	.275
Moderate/severe GCM	0.73 (0.48; 1.10)	.130
BF^a^ duration (per 1 month increase)	0.94 (0.89; 0.98)	.004
Sickness reported in the last 2 weeks: yes	0.96 (0.63; 1.46)	.841
Mother’s education: secondary or higher	0.82 (0.56; 1.22)	.332
Exposure to household tobacco smoke	1.12 (0.75; 1.66)	.586
Mother’s age	1.03 (0.99; 1.06)	.127

Sig: *P*-value at 5% level of significance.

a BF = breastfeeding.

## Data Availability

A deidentified dataset has been uploaded to OSF at https://osf.io/6h4gs.

## References

[1] Tazinya AA, Halle-Ekane GE, Mbuagbaw LT, Risk factors for acute respiratory infections in children under five years attending the Bamenda Regional Hospital in Cameroon. BMC Pulm Med 2018;18:7. 29338717 10.1186/s12890-018-0579-7 PMC5771025

[2] Simoes EAF, Cherian T, Chow J, (eds). Disease Control Priorities in Developing Countries. 2nd edn. Washington (DC): The International Bank for Reconstruction and Development/The World Bank; 2006. 21250309

[3] Ekholuenetale M, Nzoputam CI, Okonji OC, Differentials in the prevalence of acute Respiratory infections among under-five children: An analysis of 37 sub-Saharan countries. Glob Pediatr Health 2023;10:2333794×231156715. 10.1177/2333794X231156715 PMC9940173 36814530

[4] Moon TD, Sumah I, Amorim G, Antibiotic prescribing practices for acute respiratory illness in children less than 24 months of age in Kenema, Sierra Leone: Is it time to move beyond algorithm driven decision making? BMC Infect Dis 2023;23:626. 37749485 10.1186/s12879-023-08606-0 PMC10519098

[5] Impouma B, Williams GS, Moussana F, The first 8 months of COVID-19 pandemic in three West African countries: leveraging lessons learned from responses to the 2014–2016 Ebola virus disease outbreak. Epidemiol Infect 2021;149:e258. 34493348 10.1017/S0950268821002053 PMC8712942

[6] Schluger NW, Koppaka R. Lung disease in a global context. A call for public health action. Ann Amer Thor Soc 2014;11: 407–16. 10.1513/AnnalsATS.201312-420PS 24673697

[7] Dagne H, Andualem Z, Dagnew B, Acute respiratory infection and its associated factors among children under-five years attending pediatrics ward at University of Gondar Comprehensive Specialized Hospital, Northwest Ethiopia: institution-based cross-sectional study. BMC Pediatr 2020;20:93. 32111196 10.1186/s12887-020-1997-2 PMC7047350

[8] Mir F, Ariff S, Bhura M, Risk factors for acute respiratory infections in children between 0 and 23 months of age in a periurban district in Pakistan: a matched case–control study. Front Pediatr 2022;9:704545. 35083182 10.3389/fped.2021.704545 PMC8784846

[9] Nshimiyimana Y, Zhou Y. Analysis of risk factors associated with acute respiratory infections among under-five children in Uganda. BMC Public Health 2022;22:1209. 35715771 10.1186/s12889-022-13532-y PMC9205046

[10] Dahal GP, Johnson FA, Padmadas SS. Maternal smoking and acute respiratory infection symptoms among young children in Nepal: multilevel analysis. J Biosoc Sci 2009;41:747–61. 19563695 10.1017/S0021932009990113

[11] Jedrychowski W, Flak E. Maternal smoking during pregnancy and postnatal exposure to environmental tobacco smoke as predisposition factors to acute respiratory infections. Environ Health Perspect 1997;105:302–6. 9171991 10.1289/ehp.97105302 PMC1470010

[12] Yaya S, Bishwajit G. Burden of acute respiratory infections among under-five children in relation to household wealth and socioeconomic status in Bangladesh. Trop Med Infect Dis 2019;4:1. 10.3390/tropicalmed4010036 PMC6473378 30759811

[13] Gordon SB, Bruce NG, Grigg J, Respiratory risks from household air pollution in low and middle income countries. Lancet Respir Med 2014;2:823–60. 25193349 10.1016/S2213-2600(14)70168-7 PMC5068561

[14] Raju S, Siddharthan T, McCormack MC. Indoor air pollution and respiratory health. Clin Chest Med 2020;41:825–43. 33153698 10.1016/j.ccm.2020.08.014 PMC7665158

[15] Samuels RJ, Sumah I, Alhasan F, Respiratory virus surveillance in hospitalized children less than two-years of age in Kenema, Sierra Leone during the COVID-19 pandemic (October 2020- October 2021). PLoS One 2023;18:e0292652. 37816008 10.1371/journal.pone.0292652 PMC10564235

[16] Massaquoi I, Abu A, City B, Information technology and service delivery: Government Hospital, Kenema. Glob Sci J 2023;10:2022.

[17] Scientist.com. Kenema Government Hospital. https://app.scientist.com/providers/kenema-government-hospital (27 July 2023, date last accessed).

[18] Tal A, Bavilski C, Yohai D, Dexamethasone and salbutamol in the treatment of acute wheezing in infants. Pediatrics 1983;71:13–8. 6129609

[19] Golan-Tripto I, Goldbart A, Akel K, Modified Tal score: validated score for prediction of bronchiolitis severity. Pediatr Pulmonol 2018;53:796–801. 29655288 10.1002/ppul.24007

[20] World Health Organization. Guideline: Assessing and managing children at primary health-care facilities to prevent overweight and obesity in the context of the double burden of malnutrition. 2017. Available at: https://www.who.int/publications/i/item/9789241550123 29578661

[21] Lei C, Yang L, Lou CT, Viral etiology and epidemiology of pediatric patients hospitalized for acute respiratory tract infections in Macao: a retrospective study from 2014 to 2017. BMC Infect Dis 2021;21:306. 33771128 10.1186/s12879-021-05996-x PMC7995389

[22] Loevinsohn G, Hamahuwa M, Hardick J, Respiratory viruses in rural Zambia before and during the COVID-19 pandemic. Trop Med Int Health 2022;27:647–54. 35611546 10.1111/tmi.13781 PMC9348166

[23] Ogunbayo AE, Mogotsi MT, Sondlane H, Pathogen profile of children hospitalised with severe acute respiratory infections during COVID-19 pandemic in the Free State Province, South Africa. Int J Environ Res Public Health 2022;19:16. 10.3390/ijerph191610418 PMC9408356 36012053

[24] Savitha AK, Gopalakrishnan S. Determinants of acute respiratory infections among under five children in a rural area of Tamil Nadu, India. J Family Med Prim Care 2018;7:1268–73. 30613509 10.4103/jfmpc.jfmpc_131_18 PMC6293935

[25] Alexandrino AS, Santos R, Melo C, Impact of caregivers’ education regarding respiratory infections on the health status of day-care children: a randomized trial. Fam Pract 2016;33:476–81. 27131288 10.1093/fampra/cmw029

[26] Dop MC. [Breastfeeding in Africa: Will positive trends be challenged by the AIDS epidemic?]. Sante 2002;12:64–72. 11943640

[27] Geberetsadik A, Worku A, Berhane Y. Factors associated with acute respiratory infection in children under the age of 5 years: evidence from the 2011 Ethiopia Demographic and Health Survey. Pediatric Health Med Ther 2015;6:9–13. 29388598 10.2147/PHMT.S77915 PMC5683277

[28] Ibama A, Amadi A, Oti N. Nutritional status and its association with the pattern and risk of acute respiratory infections among infants in Rivers State, Nigeria: the salient factors and way out. Int J Family Med Healthcare 2023;2:1–12.

[29] Nandasena S, Wickremasinghe AR, Sathiakumar N. Indoor air pollution and respiratory health of children in the developing world. World J Clin Pediatr 2013;2:6–15. 25254169 10.5409/wjcp.v2.i2.6 PMC4145638

[30] Chalabi DAK. Acute respiratory infection and malnutrition among children below 5 years of age in Erbil governorate, Iraq. East Mediterr Health J 2013;19:66–70. 23520908

[31] Mekebo GG, Argawu AS, Likassa HT, Factors influencing exclusive breastfeeding practice among under-six months infants in Ethiopia. BMC Pregnancy Childbirth 2022;22:630. 35941576 10.1186/s12884-022-04955-x PMC9361573

[32] Abdulla F, Hossain MM, Karimuzzaman M, Likelihood of infectious diseases due to lack of exclusive breastfeeding among infants in Bangladesh. PLoS One 2022;17:e0263890. 35171952 10.1371/journal.pone.0263890 PMC8849615

