# Supplementary material for: Acute respiratory infection and associated factors among young children presenting to hospital in Sierra Leone
Source: Int Health. Author manuscript; Available in PMC 2026 Jul 24. (PMC13396981; doi:10.1093/inthealth/ihag057)
Supplement: Supplementary Figure legend [file NIHMS2192901-supplement-Supplementary_Figure_legend.docx]

Figure 1: Total number of detected viral pathogens and patterns of viral co-infection
