# Supplementary material for: Acute respiratory infection and associated factors among young children presenting to hospital in Sierra Leone
Source: Int Health. Author manuscript; Available in PMC 2026 Jul 24. (PMC13396981; doi:10.1093/inthealth/ihag057)
Supplement: Supplementary Table 1 [file NIHMS2192901-supplement-Supplementary_Table_1.docx]

Supplementary Table 1. Modified Tal severity score used to classify acute respiratory infection severity at enrolment

| Score | Modified Tal Score | | | | |
| --- | --- | --- | --- | --- | --- |
|  | Respiratory rate (breaths/min) | | Wheezing/Crackles | O2 Saturation (room air) | Accessory respiratory muscle utilization |
|  | Age <6 months | Age ≥6 months |  |  |  |
| 0 | ≤40 | ≤30 | None | ≥95 | None (no chest in-drawing) |
| 1 | 41–55 | 31–45 | Expiration only | 92–94 | + Presence of mild intercostal in-drawing |
| 2 | 56–70 | 46–60 | Expiration and inspiration with stethoscope only | 90–91 | ++ Moderate amount of intercostal in-drawing |
| 3 | ≥71 | ≥61 | Expiration and inspiration without stethoscope | ≤89 | +++ Moderate or marked intercostal in-drawing, with presence of head bobbing or tracheal tug |

**Notes:**
A total severity score was calculated as the sum of individual domain scores (each ranging from 0–3) and categorized as ≤6 (mild), 7–9 (moderate) and 10–12 (severe), as defined in the Methods.
