# Supplementary figures and images for: Acute respiratory infection and associated factors among young children presenting to hospital in Sierra Leone
Source: Int Health. Author manuscript; Available in PMC 2026 Jul 24. (PMC13396981; doi:10.1093/inthealth/ihag057)

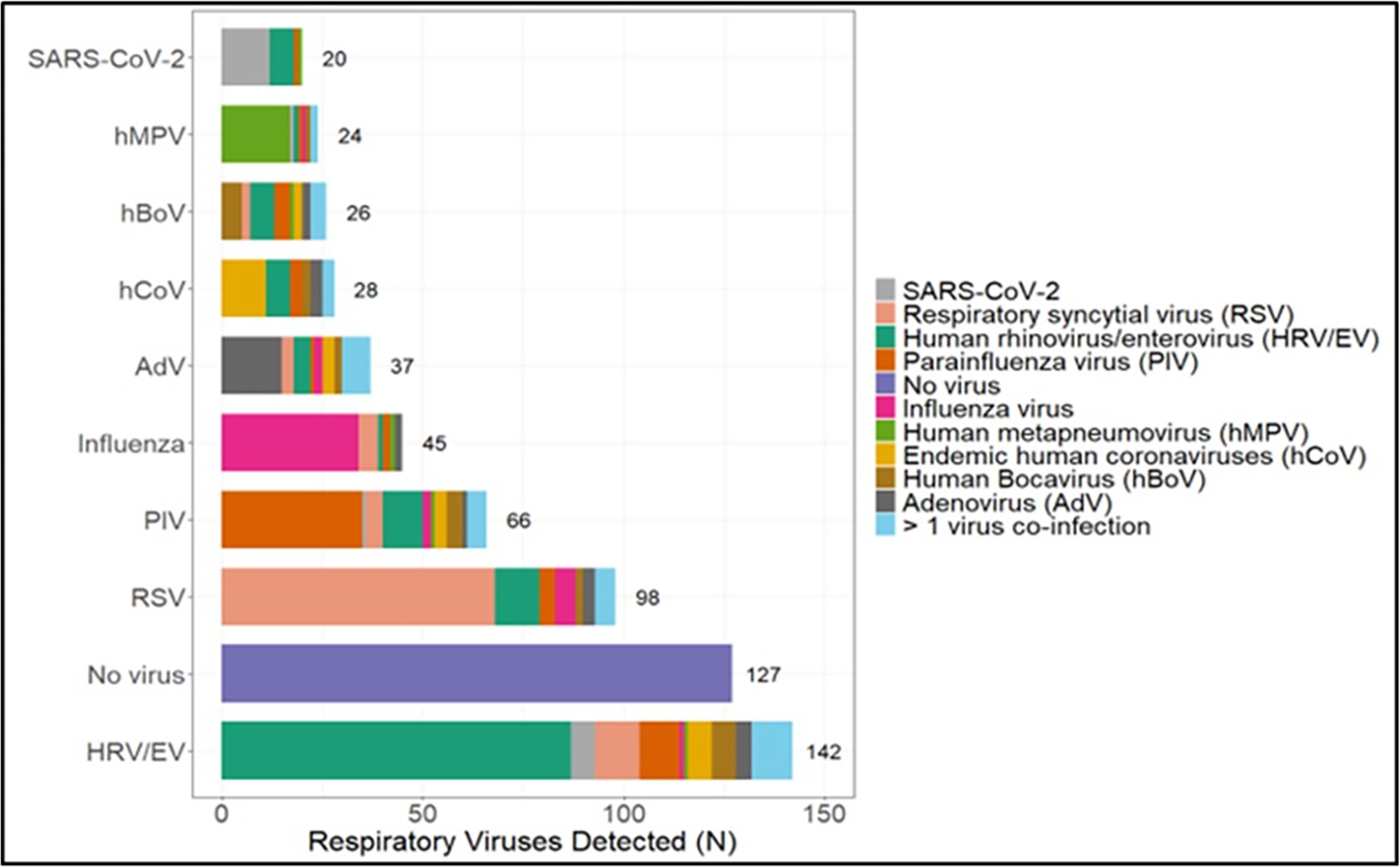

Supplement: Supplementary Figure 1 [file NIHMS2192901-supplement-Supplementary_Figure_1.docx]
